# Dynamics of Actin Cables in Polarized Growth of the Filamentous Fungus *Aspergillus nidulans*

**DOI:** 10.3389/fmicb.2016.00682

**Published:** 2016-05-09

**Authors:** Anna Bergs, Yuji Ishitsuka, Minoas Evangelinos, G. U. Nienhaus, Norio Takeshita

**Affiliations:** ^1^Department of Microbiology, Institute for Applied Bioscience, Karlsruhe Institute of TechnologyKarlsruhe, Germany; ^2^Institute of Applied Physics, Karlsruhe Institute of TechnologyKarlsruhe, Germany; ^3^Faculty of Biology, University of AthensAthens, Greece; ^4^Institute of Toxicology and Genetics, Karlsruhe Institute of TechnologyEggenstein-Leopoldshafen, Germany; ^5^Institute of Nanotechnology, Karlsruhe Institute of TechnologyEggenstein-Leopoldshafen, Germany; ^6^Department of Physics, University of Illinois at Urbana-ChampaignUrbana-Champaign, IL, USA; ^7^Faculty of Life and Environmental Sciences, University of TsukubaTsukuba, Japan

**Keywords:** actin, microtubule, *Aspergillus*, filamentous fungi, polarity

## Abstract

Highly polarized growth of filamentous fungi requires a continuous supply of proteins and lipids to the hyphal tip. This transport is managed by vesicle trafficking via the actin and microtubule cytoskeletons and their associated motor proteins. Particularly, actin cables originating from the hyphal tip are essential for hyphal growth. Although, specific marker proteins have been developed to visualize actin cables in filamentous fungi, the exact organization and dynamics of actin cables has remained elusive. Here, we observed actin cables using tropomyosin (TpmA) and Lifeact fused to fluorescent proteins in living *Aspergillus nidulans* hyphae and studied the dynamics and regulation. GFP tagged TpmA visualized dynamic actin cables formed from the hyphal tip with cycles of elongation and shrinkage. The elongation and shrinkage rates of actin cables were similar and approximately 0.6 μm/s. Comparison of actin markers revealed that high concentrations of Lifeact reduced actin dynamics. Simultaneous visualization of actin cables and microtubules suggests temporally and spatially coordinated polymerization and depolymerization between the two cytoskeletons. Our results provide new insights into the molecular mechanism of ordered polarized growth regulated by actin cables and microtubules.

## Introduction

The actin cytoskeleton plays a central role in cell morphology of eukaryotic cells. Actin filaments (F-actin), which are composed of linear polymers of G-actin subunits, generate force against the plasma membrane and also act as tracks for the movement of myosin motors. The dynamic cycles of polymerization and depolymerization of G-actin and F-actin are involved in many different key cellular processes, such as cell motility, cytokinesis, secretion and the control of cell morphology (Michelot and Drubin, 2011).

Filamentous fungi are highly polarized cells, which continuously elongate at their tips. There are three higher order F-actin structures with distinct functions in filamentous fungi: actin rings, patches, and cables (Berepiki et al., 2011). Actin rings, in cooperation with myosin II, play an essential role in septum formation (Delgado-Alvarez et al., 2010, 2014; Taheri-Talesh et al., 2012). Actin patches are peripheral punctate structures that co-localize with the endocytic machinery (Araujo-Bazan et al., 2008; Upadhyay and Shaw, 2008). The predominant localization of these patches at subapical regions suggests spatial coupling of apical exocytosis and subapical compensatory endocytosis (Penalva, 2010), in addition to endocytic recycling of polarized material (Shaw et al., 2011).

Actin cables are linear bundles of short actin filaments most likely nucleated by formins that are present at the apexes of hyphae. They are generally thought to serve as tracks for myosin V-dependent secretory vesicle transport to the tip (Taheri-Talesh et al., 2008, 2012; Berepiki et al., 2011), however, dynamic actin cables are generally very difficult to visualize. Phalloidin conjugated to fluorescent dyes has been widely used for imaging F-actin in eukaryotic cells including fungi such as budding yeast (Amberg, 1998), fission yeast (Pelham and Chang, 2001) and *Ashbya gossypii* (Walther and Wendland, 2004) but does not work in most filamentous fungi (Brent Heath et al., 2003). The “basic” growth machinery involved in the formation of actin cables, vesicle transport and exocytosis, such as formins, the polarisome, myosin V and the exocyst complex are relatively conserved among eukaryotic cells and localize to the hyphal apex of filamentous fungi (Sudbery, 2011). Before membrane fusion, the secretory vesicles accumulate at the hyphal tip in the so-called “Spitzenkörper” (Grove and Bracker, 1970; Harris et al., 2005). A Spitzenkörper is a special structure in filamentous fungi determining hyphal shape and growth direction (Bartnicki-Garcia et al., 1995; Riquelme et al., 2014). The exact composition and organization is still not completely understood, although the actin cytoskeleton is necessary for the organization of the Spitzenkörper (Sanchez-Leon et al., 2011).

Continuous supply of secretory vesicles from the hyphal cell body to the hyphal tip is essential for cell wall and cell membrane extension. Besides actin cables, microtubules and their corresponding motor proteins are involved in the secretion process (Steinberg, 2011; Egan et al., 2012; Takeshita et al., 2014). Microtubules are important for the distribution of nuclei and other organelles and serve as tracks for endosomes and other vesicles, thus they are necessary for rapid hyphal growth (Horio and Oakley, 2005). In *Aspergillus nidulans*, hyphal growth is immediately stopped if the integrity of the actin cytoskeleton is disturbed (Torralba et al., 1998). The vesicle delivery to the apical plasma membrane likely depends on the cooperation of actin and microtubule-dependent motors (Zhang et al., 2011; Schuster et al., 2012; Taheri-Talesh et al., 2012; Pantazopoulou et al., 2014). The coordinated organization of the actin and microtubule cytoskeletons is a crucial step to establish and maintain polarity (Chang and Peter, 2003; Basu and Chang, 2007; Li and Gundersen, 2008).

Specific markers for actin cables, such as Lifeact and tropomyosin, were developed and used to visualize actin cables in filamentous fungi (Taheri-Talesh et al., 2008; Berepiki et al., 2010; Delgado-Alvarez et al., 2010). Lifeact, which consists of 17 amino acids from the N-terminus of Abp140p of *Saccharomyces cerevisiae*, has been shown to be a marker for F-actin binding and labeling *in vitro* as well as in yeast cells (Riedl et al., 2008). In *Neurospora crassa*, Lifeact has been used to visualize dynamic actin cables and patches (Berepiki et al., 2010; Delgado-Alvarez et al., 2010). Tropomyosin is a conserved actin filament-binding protein and regulates the interaction between actin and myosin in response to Ca^2+^ (Gunning et al., 2005). Tropomyosin has been used as a marker for actin cables in *A. nidulans* and *N. crassa* (Pearson et al., 2004; Taheri-Talesh et al., 2008; Delgado-Alvarez et al., 2010). Tropomyosin effectively decorates actin at the Spitzenkörper and occasionally long actin cables at the hyphal tip (Pearson et al., 2004; Taheri-Talesh et al., 2008). However, the exact organization and dynamics of actin cables, such as the number, length and elongation rate of actin cables have remained elusive. Here, we have investigated the dynamic behavior of actin cables in living *A. nidulans* hyphae by using tropomyosin and Lifeact. In addition, we analyzed the regulation and relation with microtubules.

## Materials and Methods

### Strains, Plasmids, and Culture Conditions

Supplemented minimal medium for *A. nidulans* was prepared as described, and standard strain construction procedures were used (Hill and Kafer, 2001). 2% of glucose was used as carbon source, 70 mM sodium nitrate and 0.9 μM ammonium molybdate were used as nitrogen source for solid media, if not stated otherwise. For liquid media, the carbon sources were 2% glucose, 2% threonine, or 2% glycerol. *A. nidulans* strains used in this study are listed in Supplementary Table S1A. Standard laboratory *Escherichia coli* strains (Top 10 F′) were used. Plasmids are listed in Supplementary Table S1B.

### Plasmid Construction

Standard DNA transformation procedures were used for *A. nidulans* and *E. coli*. For PCR experiments, standard protocols were applied using a personal Cycler (Biometra) for the reaction cycles. DNA sequencing was performed commercially (MWG Biotech). For N-terminal tagging of TpmA with GFP, the *tpmA* gene was amplified from genomic DNA with the primers TpmA_fw and TpmA_rev (the primer sets used in this study are listed in Supplementary Table S1C). The PCR fragment was digested with *Asc*I and *Pac*I then cloned into *Asc*I-*Pac*I digested pCMB17apx (for N-terminal tagging of GFP to proteins of interest expressed under the *alcA* promoter; contains *N. crassa pyr-4*; Takeshita et al., 2008), yielding pYH27. To express GFP-TpmA under the native promoter, the promoter region was amplified from genomic DNA with the primers (p)tpmA_for and (p)tpmA_rev. digested with *Eco*RI and *Kpn*I, then cloned into *Eco*RI-*Kpn*I, digested pYH27, yielding pARB7. For N-terminal tagging of TpmA with mEos-FPthermo, mEosFP*thermo* was amplified with primers Eos_KpnI_fwd and mEos_AscI_rev, digested by *Kpn*I and *Asc*I, and subcloned into *Kpn*I-*Asc*I digested pYH27, yielding pARB1. These plasmids were transformed into the TN02A3 strain (*ku70* deletion). Primary transformants were screened microscopically for fluorescence. Integration events were confirmed by Southern blotting.

Lifeact-EGFP was amplified from pEGFP-N1-Lifeact (Riedl et al., 2008) with the primers lifeact-f-kpn and GFP_sto_PacI_rev. The PCR fragment was digested by *Kpn*I and *Pac*I, and subcloned into *Kpn*I-*Pac*I digested pCMB17apx, yielding pNT52. Lifeact-mRuby was amplified from pmRFPRuby-N1-Lifeact with the primers lifeact_mRuby_f2 and lifeact_mRuby_rev_PacI. The PCR fragment was digested by *Kpn*I and *Pac*I, and subcloned into *Kpn*I-*Pac*I digested pCMB17apx, yielding pNT51. The sequence of the calcium sensor was amplified from AAV-6P-SEW-YC3.6 with the primers Cameleon-f-kpn and Cameleon-r-pac. The PCR fragment was digested by *Kpn*I and *Pac*I, and subcloned into *Kpn*I-*Pac*I digested pCMB17apx, yielding pARB10. The plasmid pNT51 was transformed into the wild type strain GR5. The plasmid pARB10 was transformed into the TN02A3 strain. Primary transformants were screened microscopically for fluorescence and by PCR for correct integration of the constructs.

### Epifluorescence Microscopy

Cells were grown either in Fluorodishes FD35-100 (World Precision Instruments) or on cover slips (Carl Roth) with minimal medium + 2% carbon source at 28°C overnight. Images were captured using an Axiophot microscope using a planapochromatic 63x/1.4 N.A. oil immersion objective lens, the ZEISS AxioCam MRM camera (ZEISS, Jena, Germany), and the HBO103 mercury arc lamp (Osram) or HXP 120 (ZEISS) possessing faster speed wavelength switching. Images were collected and analyzed using the Zen system (ZEISS).

### Photo-conversion of mEos-FP*thermo*

The images of TpmA tagged with photoconvertible mEos-FP*thermo* were acquired at room temperature on a modified inverted microscope Axiovert 200 (ZEISS) equipped with a high N.A. water immersion objective (C-Apochromat, 63x, N.A. 1.2, ZEISS). We employed three diode pumped solid-state lasers, with wavelengths 561 nm (Cobolt Jive 561-150, Cobolt, Sweden), 473 nm (LuxX 473-100, Omicron Laserage, Rodgau-Dudenhofen, Germany) and 405 nm (Stradus 405-250, Vortran Laser Technology, Sacramento, CA, USA) for excitation and photo activation of the fluorophores. The laser sources were combined via dichroic mirrors and guided through an AOTF (AOTFnC- 400.650, AA Opto-Electronic, Orsay, France) to control the laser intensities at the sample. Cells were incubated at 28°C overnight in a chambered cover glass. For the pulse-chase experiment, a small fraction of photoconvertible fluorescent proteins at the tip region of the cell were locally converted from their green to their red emitting forms using focused 405 nm laser illumination (1.9 μm^2^, 163 W/cm^2^, 1 s) and excited by 561 nm illumination (50 W/cm^2^). After passing through the excitation dichroic (z 405/473/561/635, AHF, Tübingen, Germany), fluorescence emission was filtered by a 607/50 band-pass filter (AHF) and recorded with a back-illuminated EMCCD camera (Ixon Ultra 897, Andor Technology, Belfast, UK) at 200 ms/frame. Acquired images were processed by ImageJ software.

### Super Resolution Microscopy

For confocal imaging, samples were observed using an Axio Observer Z.1/LSM880 confocal microscope (Carl ZEISS microscopy) equipped with an oil-immersion objective (Plan Apochromat 63x/1.4; Carl ZEISS). Excitation laser wavelengths were 488 for EGFP and 561 nm for mCherry. Fluorescence signals were detected using Airyscan detector with SR (super resolution) mode (Weisshart, 2014). Images were collected by ZEN software (ZEISS).

### Spinning Disk Confocal Microscopy

Cells expressing GFP-TpmA and mCherry-TubA were grown in 8 well glass bottom slides (ibidi) with minimal medium + 2% glycerol at 28°C overnight. Images were captured using a conventional AxioObserver Z1 inverted microscope employing a Plan-Apochromat 63x/1.40 N.A. oil Ph3 M27 (ZEISS) objective lens, a ZEISS Multi Laser module with a 488 Diode Laser and a 561nm OPSL Laser and a spinning disk module CSU-X1M 5000. Image capture was carried out by Evolve 512 Camera (Photometrics). Images were collected and analyzed using Zen software (ZEISS).

### Quantitative Real-Time PCR

For RNA isolation mycelium was collected after 1 day growth in minimal medium, shock frozen in liquid nitrogen and crushed with glass beads in RB buffer (OMEGA bio-tek) using a RetschMM200 mixer mill. RNA was extracted with the E.Z.N.A Fungal RNA Mini Kit (OMEGA bio-tek) following the manufacturer’s protocol. For DNA digestion the Ambion Turbo DNA Free Kit (Invitrogen) was used. For quantitative real time PCR the Bioline SensiFast SYBR and Fluorescein One Step Kit were used according to the manufacturer’s protocol and were analyzed in an iCycler iQ detection system (Bio-Rad). Three technical and three biological replicates were performed. Histone H2B was used as a house-keeping gene and was amplified with H2B-FW-qRT-PCR and H2B-Rev-qRT-PCR. The other templates were amplified by using the primer set qRT_GFP_fw and qRT_GFP_rev. With the ΔΔCt- method the relative expressions were calculated.

## Results

### Tropomyosin TpmA as a Marker for Dynamic Actin Cables

**FIGURE 1:**
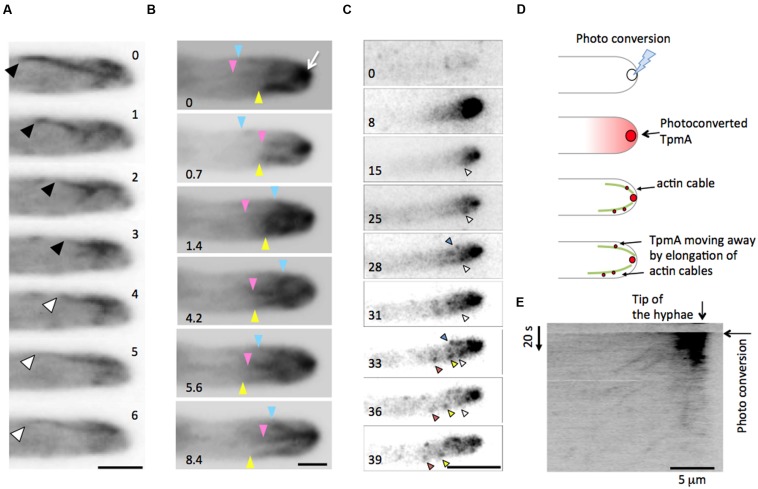
**Tropomyosin TpmA as a marker for dynamic actin cable. (A)** Elongation and shrinkage of an actin cable visualized by GFP-TpmA. Wide-field image sequence of a dynamic actin cable at the hyphal tip of *Aspergillus nidulans* expressing GFP-TpmA. Black arrowheads indicate shrinkage of the actin cable, whereas white arrowheads indicate elongation of the actin cable. The elapsed time is given in seconds. Scale bar: 2 μm. **(B)** Three actin cables showed elongation and shrinkage in an independent manner. Blue, pink, and yellow arrowheads indicate the minus ends of the actin cables. The elapsed time is given in seconds. Scale bar: 1 μm. **(C–E)** Tracking TpmA within the actin cable visualized by using the photoconvertible fluorescent protein mEosFP*thermo*. **(C)** Image sequences of photoconverted mEosFP*thermo*-TpmA. Photoconversion was done at *t* = 7 s for the duration of 1 s. The differently colored arrowheads indicate the movement of mEosFP*thermo*-TpmA. The elapsed time is given in seconds. Scale bar: 5 μm. **(D)** Schematic representation of the pulse-chase experiment. mEosFP*thermo*-TpmA at the hyphal tip was photoconverted to its red form by focused 405-nm light. Actin cables are elongated at the plus end close to the plasma membrane by formin adding G-actin to the plus end. The photoconverted red signals moved away from the hyphal tip by elongation of actin cables at the plus end. **(E)** Kymograph from image sequences **(C)**. The profile shows that photoconverted mEosFP*thermo*-TpmA moves linearly in time, which is indicative of the active elongation of an actin cable. Vertical scale: 20 s, horizontal scale bar: 5 μm.

In order to investigate the dynamics of actin cables in *A. nidulans*, strains expressing GFP fused to the N-terminus of the tropomyosin TpmA (GFP-TpmA) under the native or adjustable *alcA* promoter were constructed. Besides the native TpmA, one copy of the DNA fragment for GFP-TpmA expression was inserted into the native locus (see Materials and Methods). The strains showed no obvious effect on colony growth, spore formation and morphology of hyphae (**Supplementary Figure S1A**). Under de-repressed conditions of the *alcA* promoter (glycerol as carbon source), the expression level of *gfp*-*tpmA* was equal to that under the native *tpmA* promoter (**Supplementary Figure S1B**). The strain showed the identical hyphal morphology as the wild type (**Supplementary Figure S1A**). Under these conditions, GFP-TpmA visualized dynamic actin cables with cycles of elongation and shrinkage (**Figures 1A,B**) and decorates actin at the Spitzenkörper (**Figure 1B** arrow), which is consistent with previous reports (Pearson et al., 2004; Taheri-Talesh et al., 2008). However, no labeling of actin patches was observed. The strain expressing GFP-TpmA under the native promoter also showed similar actin cables (data not shown). In addition to hyphal tips, GFP-TpmA localized to septation sites (see below).

The actin cables were observed at most of the hyphal tips (86%, *n* = 100) by epifluorescence microscopy with a wide focus depth (covering 0.4 μm). To quantify the dynamic behavior, we took image sequences with 100–200 ms intervals (total 30–60 s). The image data of actin cables clearly covered within the focus were used for quantification. The elongation and shrinkage rates of actin cables were similar, 0.58 ± 0.03 μm/s (mean ± SEM, *n* = 76) and 0.59 ± 0.03 μm/s (mean ± SEM, *n* = 100), respectively (**Table 1** and **Supplementary Figure S2**). The length of the actin cables was determined as 2.9 ± 1.2 μm (mean ± SD, *n* = 108) just before they disassembled again. These results indicate that GFP-TpmA is a proper marker to visualize dynamic actin cables, however, we cannot exclude the possibility that GFP-TpmA decorates actin cables but does not represent the whole actin cable population. Due to their dynamic behavior, we might not be able to visualize some of the actin cables at all times.

**Table 1:** Dynamic behavior of actin cables.

	GFP-TpmA	Lifeact-GFP	Lifeact-GFP
	Glycerol	Glycerol	Thr + 0.01% Glucose
Elongation rate (μm/s),	0.58 ± 0.03	0.09 ± 0.03	0.43 ± 0.08
(mean ± SEM)	(*n* = 76)	(*n* = 37, *p* < 0.01^∗^)	(*n* = 13, *p* = 0.06^∗^, *p* < 0.01^∗∗^)
Shrinkage rate (μm/s),	0.59 ± 0.03	0.1 ± 0.03	0.61 ± 0.1
(mean ± SEM)	(*n* = 100)	(*n* = 43, *p* < 0.01^∗^)	(*n* = 13, *p* = 0.8^∗^, *p* < 0.01^∗∗^)
Length before disassembly (μm)	2.9 ± 1.2	9.4 ± 5.9	4.0 ± 1.0
(mean ± SD)	(*n* = 108)	(*n* = 20, *p* < 0.01^∗^)	(*n* = 13, *p* < 0.01^∗^, *p* < 0.01^∗∗^)

^∗^*Single asterisks represent statistically significant differences compared to GFP-TpmA.*^∗∗^*Double asterisks represent statistically significant differences compared to Lifeact-GFP in Gly.*

Overexpression of *tpmA* under the *alcA* promoter with 2% threonine as carbon source had no obvious effect on colony growth, spore formation and the morphology of hyphae (**Supplementary Figure S1**). The fluorescence image showed an actin cable pattern as observed in glycerol medium, but with a significantly higher cytoplasmic background (**Supplementary Figure S1E**). Under the overexpression condition, elongation and shrinkage rates of actin cables were reduced to 0.28 ± 0.03 μm/s (mean ± SEM, *n* = 11, *p* < 0.01) and 0.37 ± 0.04 μm/s (mean ± SEM, *n* = 17, *p* < 0.01), respectively, in comparison to glycerol medium.

To track the localization of TpmA within a polymerizing actin cable, GFP was replaced with the photoconvertible fluorescent protein mEosFP*thermo*, a thermostable, monomeric variant of the green-to-red photoconverting fluorescent protein, EosFP, to perform a pulse-chase experiment (Nienhaus et al., 2005; Wiedenmann and Nienhaus, 2006; Fuchs et al., 2010). mEosFP*thermo*-TpmA at the hyphal tip was locally photoconverted to its red form by tightly focused 405-nm irradiation (**Figure 1C** at 8 s). The red signals from photoconverted mEosFP*thermo*-TpmA can be detected in the cytosol as a diffuse background and within the dynamic actin cable as punctate spots (**Figures 1C,D**, see Materials and Methods). The actin cable is not stained evenly because not all mEosFP*thermo*-TpmA proteins had been photoconverted. The red signals slowly moved away from the tip of hyphae (**Figure 1C**). The kymograph image of the signals showed a clear linear movement away from the tip (**Figure 1E**). This is consistent with the mechanism of actin polymerization, where new G-actin molecules are incorporated at the plus end of the actin cable.

### Expression Level of Lifeact Affects the Dynamics of Actin Cables

**FIGURE 2:**
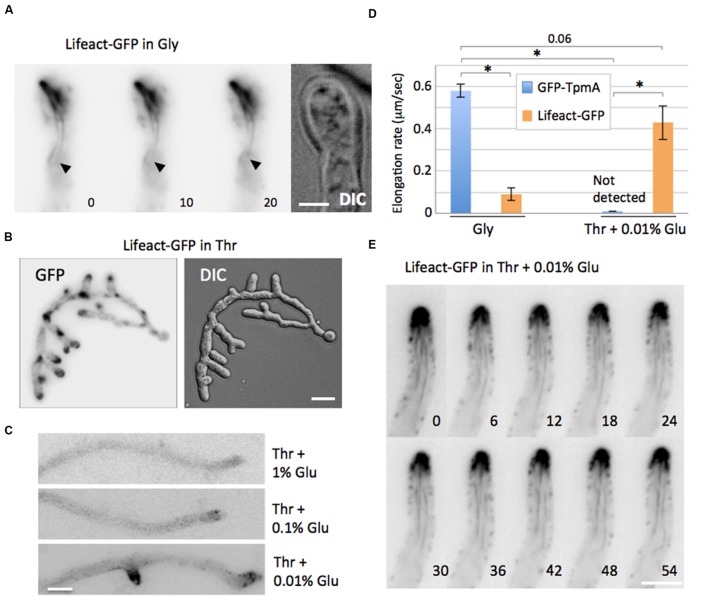
**Expression level of Lifeact affects the dynamics of actin cables. (A)** Actin cables visualized by Lifeact-GFP were stable. Lifeact-GFP was expressed under the *alcA* promoter in glycerol containing medium. The arrowheads indicate the position of the end of one actin cable. The elapsed time is given in seconds. Scale bar: 2 μm. **(B)** Elongation rates of actin cables quantified in the strains expressing GFP-TpmA (blue) or Lifeact-GFP (orange). GFP-TpmA or Lifeact-GFP were expressed under the *alcA* promoter in 2% glycerol medium (left) or 2% threonine and 0.01% glucose medium (right). The data are expressed as mean ± SEM (*n* = 76, 37, 10, and 13, respectively). Asterisks represent statistically significant difference, *p* < 0.01. **(C)** Overexpression of Lifeact-GFP. The strain expressing Lifeact-GFP was grown in medium containing 2% threonine. Scale bar: 5 μm. **(D)** Different expression levels of Lifeact-GFP under carbon catabolite repression. The strain expressing Lifeact-GFP was grown in medium containing 2% threonine plus 1, 0.1, or 0.01% glucose. Scale bar: 2 μm. **(E)** Wide-field image sequences of actin cables visualized by Lifeact-GFP in medium containing 2% threonine plus 0.01% glucose. The elapsed time is given in seconds. Scale bar: 5 μm.

To investigate the effect of Lifeact on the dynamics of actin cables, a strain expressing Lifeact tagged with GFP at the C-terminus ectopically under the *alcA* promoter was constructed. Under de-repressing conditions in minimal medium with 2% glycerol as carbon source, the colony size was the same as for the wild type (**Supplementary Figure S1A**), but the hyphae sometimes exhibited an abnormal morphology with swollen tips (**Figure 2A**). Lifeact-GFP showed actin cables and actin patches at all hyphal tips (*n* = 100). The actin cables visualized by Lifeact-GFP showed a mesh-like structure that was significantly less dynamic than that of GFP-TpmA. The elongation rate of actin cables visualized by GFP-TpmA was 0.58 ± 0.03 μm/s (mean ± SEM, *n* = 76), whereas that of Lifeact-GFP was 0.09 ± 0.03 μm/s (mean ± SEM, *n* = 37), decreasing to 16% of GFP-TpmA (**Figure 2B**). The average length of actin cables visualized by Lifeact-GFP was three times the one of GFP-TpmA (**Table 1** and **Supplementary Figure S2**).

Overexpression of Lifeact-GFP in minimal medium with 2% threonine as carbon source caused abnormal hyphal morphology with more branching and swollen tips (**Figure 2C**). Stable mesh-like structures were observed at the hyphal tips. In this condition, the wild type strain did not show such abnormal hyphal morphology with more branching and swollen tips (data not shown). These results suggest that the expression level of Lifeact-GFP affects the dynamics of actin cables. To further explore this idea, Lifeact-GFP was expressed under different carbon source conditions, 2% threonine with 1, 0.1, or 0.01% glucose. The expression of Lifeact-GFP was expected to decrease by adding glucose. Using 2% threonine and 1 or 0.1% glucose, the hyphae looked similar to the wild type but the GFP signal was hardly detectable (**Figure 2D**).

When Lifeact-GFP was expressed with 2% threonine and 0.01% glucose, the GFP signal of actin cables appeared at hyphal tips and the hyphae showed a normal morphology (**Figure 2C**), and the expression level was 44% of that in minimal medium with 2% glycerol (**Supplementary Figure S1C**, overnight in shaking culture). The GFP signal intensity in the hyphal tip region was less than 10% of that in minimal medium with 2% glycerol (**Supplementary Figure S1D**), and the actin cables decorated by Lifeact-GFP restored their dynamic behavior (**Figures 2B,E**). The elongation rates of actin cables visualized by Lifeact-GFP were restored up to 0.43 ± 0.08 μm/s (mean ± SEM, *n* = 13). Although the elongation and shrinkage rates of actin cables labeled by Lifeact-GFP did not show significant differences compared to GFP-TpmA, the average length of those actin cables was 1.4 times greater than that of GFP-TpmA labeled actin (**Table 1** and **Supplementary Figure S2**). Since GFP-TpmA expressed under the *alcA* promoter did not show clear actin cables under the same conditions (2% threonine and 0.01% glucose; **Supplementary Figure S1F**), the elongation rate of actin cables could not be measured (**Figure 2B**). These results suggest that higher concentrations of Lifeact-GFP reduce the dynamics of actin cables and cause more stable actin cable structures. On the other hand, lower concentrations of Lifeact-GFP showed comparable dynamics of actin cables to GFP-TpmA, suggesting Lifeact expressed at lower level is suitable as an actin cable marker in *A. nidulans*. The wild type and the strains expressing GFP-TpmA showed no such abnormal morphology under all tested conditions (**Supplementary Figures S1E,F**).

### Effect of Lifeact on the Actin Structures Visualized by GFP-TpmA

**FIGURE 3:**
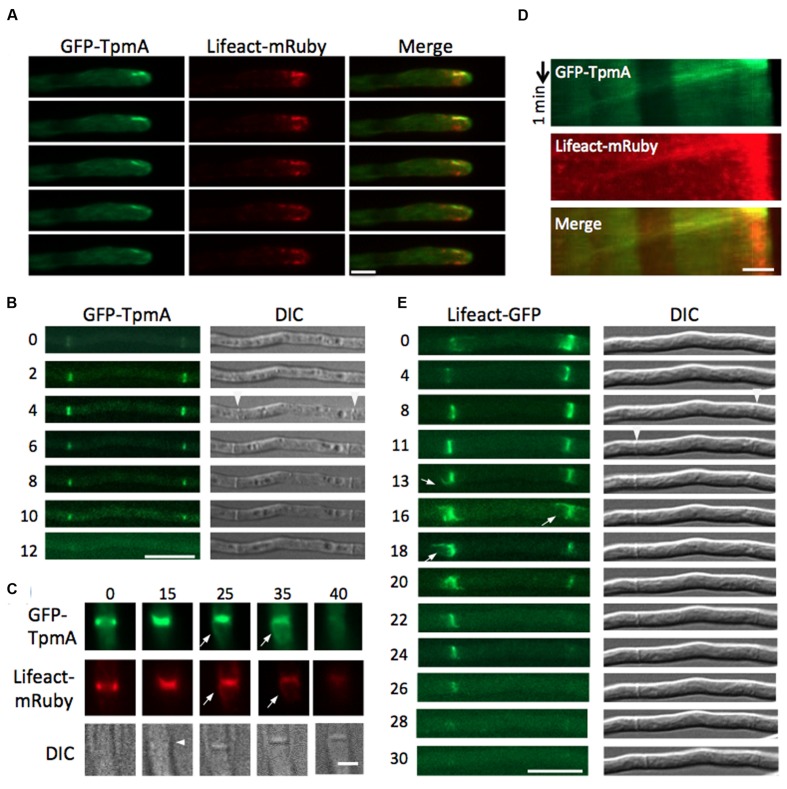
**Effect of Lifeact-mRuby on the actin structures visualized by GFP-TpmA. (A)** GFP-TpmA co-localized with Lifeact-mRuby along stable actin cables. The strain expressing GFP-TpmA and Lifeact-mRuby was grown in glycerol containing medium. The elapsed time is given in seconds. Scale bar: 2 μm. **(B)** Kymograph of actin cable dynamics from image sequences **(A)**. Vertical arrow: 1 min, horizontal scale bar: 2 μm. Wide-field image sequences of actin rings visualized by GFP-TpmA **(C)** or Lifeact-GFP **(D)** or GFP-TpmA and Lifeact-mRuby **(E)** in 2% glycerol containing medium. White arrowheads indicate septa recognized by DIC. The elapsed time is given in seconds. **(C,D)** Scale bar: 10 μm. **(E)** GFP-TpmA and Lifeact-mRuby localized to the actin ring prior to visibility of the septum in the DIC channel. Scale bar: 2 μm.

To confirm the effect of Lifeact, we constructed a strain expressing GFP-TpmA and Lifeact fused to the bright red fluorescent protein mRuby (Lifeact-mRuby) under the control of the *alcA* promoter (Kredel et al., 2009). Under de-repressed conditions, the strain expressing only GFP-TpmA showed dynamic actin cables (**Figure 1**), whereas the strain expressing only Lifeact-GFP exhibited more stable actin cables (**Figures 2A,B**; **Table 1**). When the strain expressing both GFP-TpmA and Lifeact-mRuby was grown under de-repressed conditions, GFP-TpmA and Lifeact-mRuby co-localized along actin cables (**Figures 3A,B**, **Supplementary Figure S1G**), which did not show clear dynamics as observed with GFP-TpmA only. The results suggest that both actin markers visualize the same actin cables and that Lifeact-mRuby stabilizes these actin cables resulting in a decrease in the dynamics of GFP-TpmA visualized actin cables.

Roles and localization of the actin cytoskeleton on septum formation had been analyzed previously (Harris, 2001; Delgado-Alvarez et al., 2014). The shrinkage of the actomyosin ring, which is formed by actin filaments and myosin II, is associated with septum formation (Taheri-Talesh et al., 2012). In order to see if there is a stabilizing effect of Lifeact on the actin structures during septum formation, we compared the localization change of actin during septum formation by using GFP-TpmA and Lifeact-GFP (**Figures 3C,D**). Both GFP-TpmA and Lifeact-GFP localized to the actin ring prior to visibility of the septum in the DIC channel (**Figures 3C,D**). After the septum was clearly discernible by DIC, the actin ring visualized by GFP-TpmA started to shrink and finally disappeared within approximately 10 min. In contrast, the actin ring visualized by Lifeact-GFP did not shrink for a while (5–10 min), then shrunk and finally disappeared approximately 15–20 min after the septum was observed by DIC. These results indicate that Lifeact-GFP localizes at the actin ring longer than GFP-TpmA. The difference between TpmA and Lifeact during septum formation could be due to a stabilizing effect of Lifeact on actin filaments. In the strain expressing both GFP-TpmA and Lifeact-mRuby, GFP-TpmA stayed at the septation site longer than 20 min after the septum was visualized by DIC (**Figure 3E**). These results also support the idea that Lifeact-mRuby stabilizes the actin cables, resulting in a decrease in the dynamics of GFP-TpmA visualized actin cables.

### Localization of Actin Cables with the Cell End Marker and Microtubules

**FIGURE 4:**
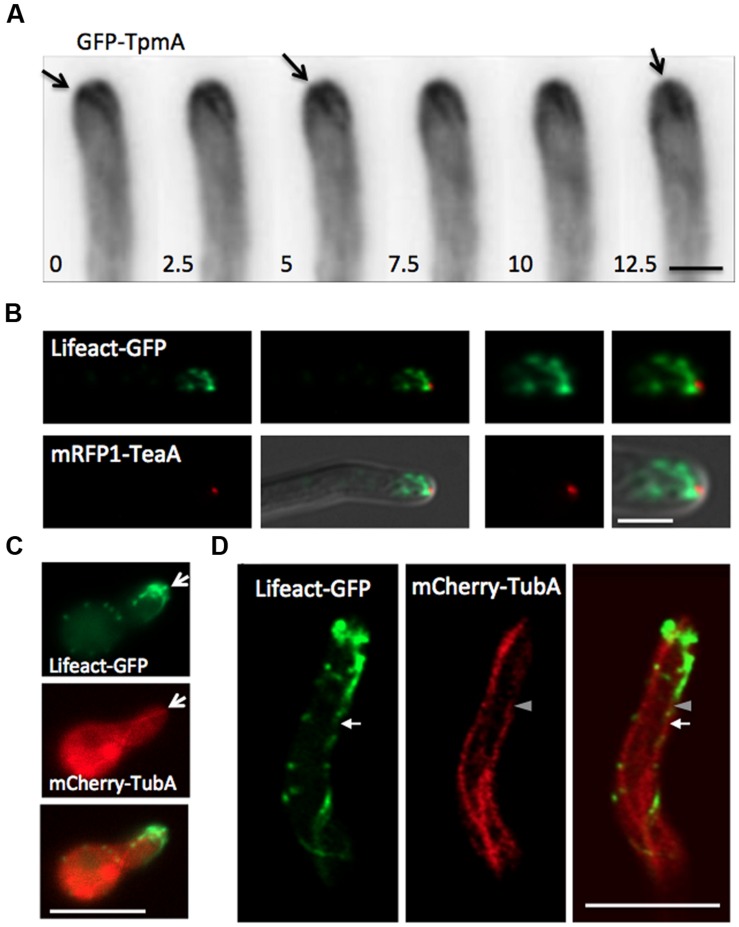
**Localization of actin cables with cell end marker or microtubules. (A)** Wide-field image sequences of an actin cable by GFP-TpmA. The plus end, visualized by GFP-TpmA, moved along the apical membrane. Arrows indicate the plus end of the actin cable moving along the apical membrane. The elapsed time is given in seconds. Scale bar: 2 μm. **(B)** The strain expressing Lifeact-GFP under the *alcA* promoter and mRFP1-TeaA under the native promoter was grown in 2% glycerol containing medium. Scale bar: 2 μm. **(C,D)** Localization of actin cables by Lifeact-GFP and microtubules by mCherry-TubA. Super-resolution microscopy image was constructed by Airyscan (ZEISS; **D**). Scale bars: 2 μm.

Actin filaments are polymerized at the plus ends close to the plasma membrane by formins (Goode and Eck, 2007). It was shown before that the cell end marker TeaA and the formin SepA colocalize at the hyphal tip by indirect interaction (Takeshita et al., 2008; Higashitsuji et al., 2009). In addition, TeaA is necessary for SepA to localize stably at the apex of hyphal tips (Takeshita et al., 2013). We could observe the plus ends of actin cables at the hyphal tip cortex and found that the plus end sometimes moved along the apical membrane (**Figure 4A**). To investigate the relation of localization between TeaA and the plus ends of actin cables, we constructed a strain expressing Lifeact-GFP and mRFP1-TeaA. Actin cables visualized by Lifeact-GFP originated close to the mRFP1-TeaA accumulation site at the hyphal tip (**Figure 4B**), supporting the idea that actin cables are organized from polarity sites where cell end markers accumulate. Although, the cell end marker regulates the localization of formin along the apical membrane (Takeshita et al., 2013), resulting in the localization of actin cable plus end, it is not yet clear that the cell end marker is involved in the regulation of formin activity and actin cable formation.

TeaA localizes at microtubule plus ends and is transported to the hyphal tip by growing microtubules (Fischer et al., 2008; Takeshita and Fischer, 2011). To analyze the interrelation of actin cables and microtubules, we constructed a strain expressing Lifeact-GFP and mCherry-TubA (α-tubulin), and compared their localization (**Figures 4C,D**). The microtubule plus end reached the apical cortex, where it co-localized with the origin of the actin cable meshwork (**Figure 4C**, arrows). The strain was observed by a laser-scanning confocal microscope that features a proprietary detector called an Airyscan (ZEISS) for super resolution imaging (**Figure 4D**; see Materials and Methods; Weisshart, 2014). The actin cable originating from the hyphal tip reached to the microtubule plus end behind the hyphal tip (arrow and arrowhead). There appeared a short-range overlap between the microtubule plus end and the minus end of the actin cable.

### Related Dynamics between Actin Cables and Microtubules

**FIGURE 5:**
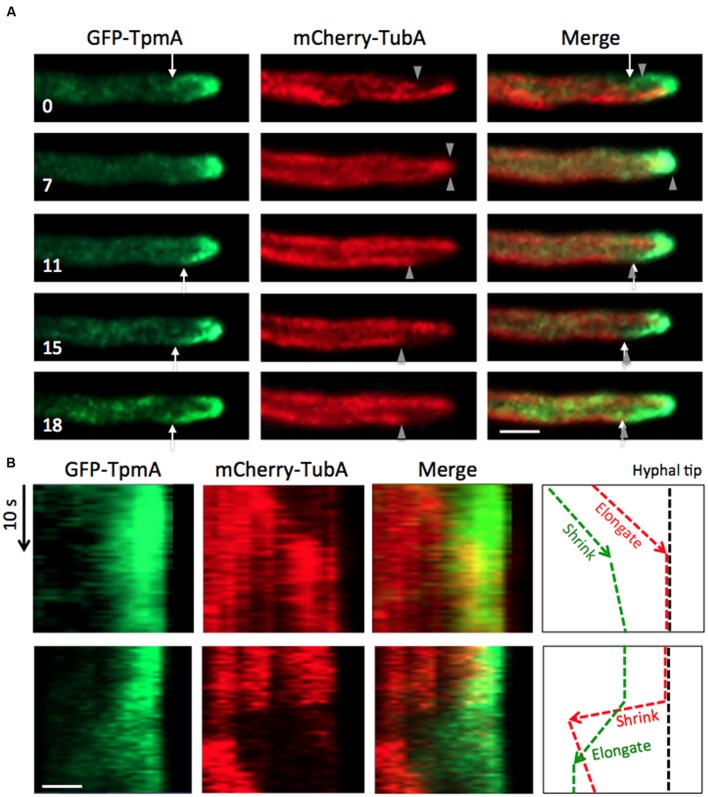
**Coordinated elongation between actin cables and microtubules at hyphal tips. (A)** Image sequences with approximately 200 ms interval taken via spinning-disk confocal microscopy of actin cables visualized by GFP-TpmA and microtubules visualized by mCherry-TubA. Arrows indicate the ends of actin cables. Arrowheads indicate the plus ends of microtubules. The elapsed time is given in seconds. Scale bar: 5 μm. **(B)** Kymograph of actin cable and microtubule dynamics from image sequences **(A)**. Vertical arrow: 10 s, horizontal scale bar: 2 μm.

**FIGURE 6:**
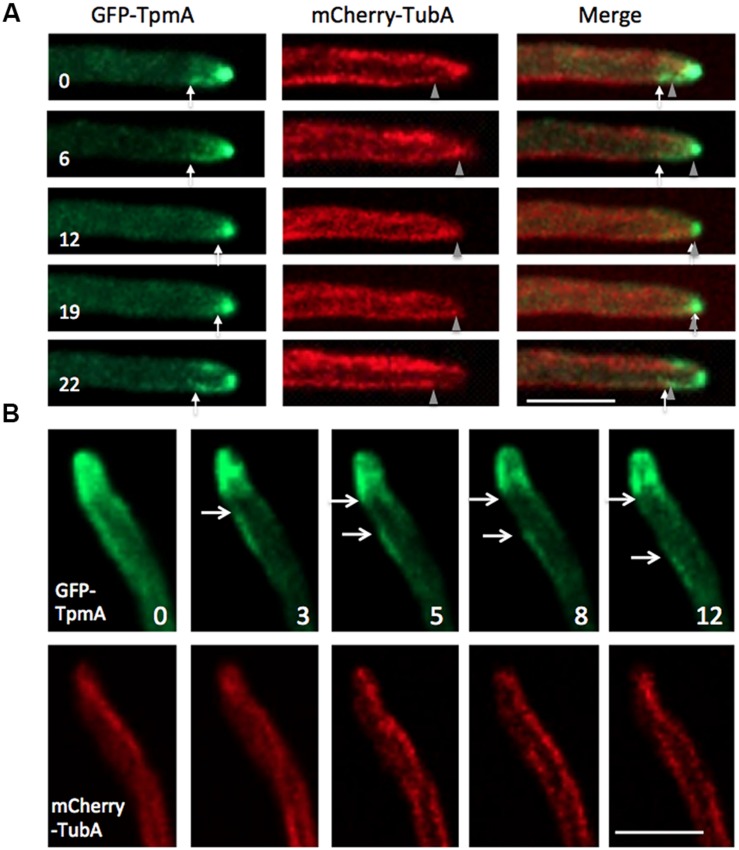
**Related dynamics between actin cables and microtubules. (A,B)** Image sequences with approximately 200 ms interval taken via spinning-disk confocal microscopy of actin cables visualized by GFP-TpmA and microtubules visualized by mCherry-TubA. Arrows indicate the ends of actin cables. Arrowheads indicate the plus ends of microtubules. The elapsed time is given in seconds. Scale bar: 5 μm.

A functional relation between the actin and microtubule cytoskeletons is crucial for polarized growth of hyphae (Takeshita et al., 2014). To investigate their interrelation, we constructed a strain expressing GFP-TpmA and mCherry-TubA and analyzed the dynamics by spinning-disk confocal microscopy to obtain a higher time resolution of dual-color images (**Figures 5** and **6**; see Materials and Methods). Microtubules showed dynamic behavior with cycles of polymerization and disassembly. Around the hyphal tip, microtubules elongated toward the tip until they reached the apical cortex, stayed there for a certain duration and then rapidly retracted. In **Figure 5A**, one microtubule (lower) reached the apical cortex at *t* = 0. While the other microtubule (upper, arrowhead) elongated and reached the apical cortex at *t* = 7 s, the actin cable (*t* = 0, arrow) disappeared at *t* = 7 s. The one of the microtubules (lower, *t* = 11 s, arrowhead) shrunk and the plus end stayed a few micrometers behind the hyphal tip (*t* = 15, 18 s, arrowheads). The other actin cable was formed immediately after shrinkage of the microtubule (*t* = 11–18 s, arrows). The minus end (pointed end) of the actin cable reached the plus end of the microtubule (*t* = 15, 18 s). There appeared a short-range overlap between the microtubule plus end and the minus end of the actin cable, as in the super resolution image in **Figure 4D**. The dynamics was confirmed by the kymographs (**Figure 5B**). While the microtubule elongated and reached the hyphal tip, the actin cable shrank (**Figure 5B**, upper). After shrinkage of the microtubule, the actin cable elongated immediately (**Figure 5B**, lower). The shrinkage rate of the microtubule appeared greater than the elongation rate of the actin cable, suggesting no direct interaction between actin cables and microtubules during the microtubular catastrophe.

On the other hand, when the microtubule elongated to the hyphal tip (**Figure 6A**, *t* = 0, 6 s, arrowheads), the microtubule plus end appeared to move along the actin cable. When the microtubule plus end reached the tip cortex, the actin cables shrunk back toward the hyphal tip (**Figure 6A**, *t* = 12 s). While the microtubule plus end touched the apical cortex, there were no actin cables formed from the hyphal tip (**Figure 6A**, *t* = 12–19 s). When the microtubule shrunk a few micrometers, the new actin cable started forming from the hyphal tip to the microtubule plus end (**Figure 6A**, *t* = 22 s). Occasionally, long actin cables divided into two (**Figure 6B**). The upper part of the cleaved actin cable at the hyphal tip shrunk, and the other part of the cleaved actin cable was transported toward the hyphal body along microtubules.

To examine the temporal relation between actin cable and microtubule polymerization, we quantified the frequency of actin cable disassembly (per hypha and min) from the images of GFP-TpmA (6.7 ± 2.5 min^–1^, mean ± SD, *n* = 12). The frequency of microtubules reaching the hyphal tip per minute was quantified before under the same conditions (5.8 ± 1.7 min^–1^, mean ± SD, *n* = 20; Pöhlmann et al., 2014). The disassembly frequency of actin cables and the frequency of microtubules reaching the hyphal tip did not show significant differences (**Supplementary Figure S2D**).

### Actin Cable Formation in Different Situations

**FIGURE 7:**
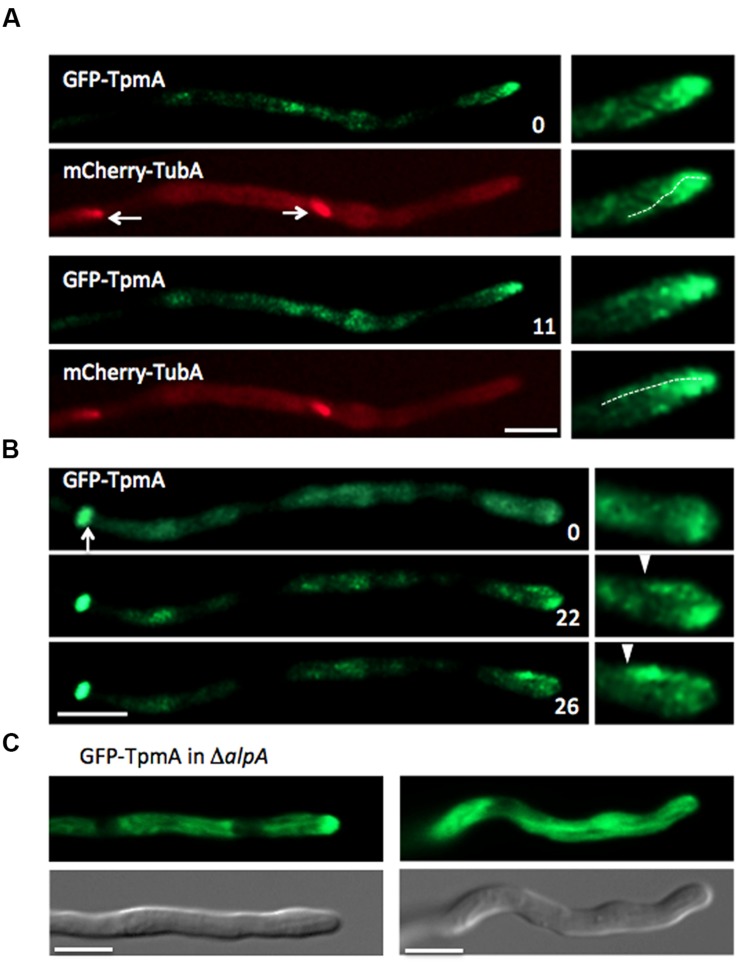
**Actin cable formation in mitosis, septum formation or D***alpA*** strain. (A)** Actin cables visualized by GFP-TpmA at the hyphal tip during mitosis (dotted lines, right). Spindles were visualized by mCherry-TubA (arrows). The elapsed time is given in seconds. Scale bar: 5 μm. **(B)** Actin cables visualized by GFP-TpmA at the hyphal tip during septum formation. The forming septum is shown by an arrow. The ends of actin cables are marked by arrowheads (right). The elapsed time is given in seconds. Scale bar: 5 μm. **(C)** The D*alpA* strain expressing GFP-TpmA under the *alcA* promoter was grown in 2% glycerol containing medium. Scale bars: 5 μm.

In budding and fission yeasts, the organization of the actin cytoskeleton is regulated by the cell cycle (Mishra et al., 2014). We investigated the behavior of the actin cytoskeleton during mitosis in *A. nidulans* in the strain expressing GFP-TpmA and mCherry-TubA. During mitosis, spindles were visualized by mCherry-TubA (**Figure 7A**). Dynamic actin cables visualized by GFP-TpmA were still observed at the hyphal tip during mitosis (**Figure 7A**). The actin cable dynamics was similar to that of interphase cells. This is consistent with the result that the growth speed of hyphae is not obviously affected between interphase and mitosis (Riquelme et al., 2003; Horio and Oakley, 2005). During septum formation, actin rings formed at the septation sites (**Figures 3C,D**). Even in the presence of an actin ring at the tip compartment, actin cables could still be observed at the tips of hyphae (**Figure 7B**).

Since coordinated elongation between actin cables and microtubules was suggested (**Figures 5** and **6**), we examined the localization of actin cables in the *alpA* mutant, which shows a defect in microtubule organization (Enke et al., 2007). AlpA is a member of the XMAP215 protein family and acts as a microtubule polymerase by addition of tubulin dimers to the growing plus end (Brouhard et al., 2008; Takeshita et al., 2013). The deletion of *alpA* results in a severe phenotype, including reduced colony size and compact growth, as well as curved growing hyphae and increased branching (Enke et al., 2007). In the *alpA* deletion strain, only one or two thick microtubule filaments were observed in one hyphal compartment between two septa or between the hyphal tip and the next septum (Enke et al., 2007). This microtubule filament showed no obvious dynamics (Takeshita et al., 2013). In the *alpA* deletion strain, one long actin cable decorated by GFP-TpmA was occasionally observed (<10 μm; **Figure 7C**). The long actin cable appeared to be more stable and did not show clear cycles of elongation and shrinkage, which was never observed in the wild type control. This may be due to a colocalization with the stable microtubule filament that was observed in the *alpA* deletion strain.

## Discussion

The comparison between TpmA and Lifeact under different expression levels revealed that the expression level of Lifeact is crucial to visualize intact actin cables and not interfere with their function. Low-level expression of Lifeact-GFP showed comparable dynamics of actin cables as GFP-TpmA (**Table 1** and **Supplementary Figure S2**), suggesting Lifeact expressed at lower levels and TpmA are suitable as actin cable marker in *A. nidulans*. On the other hand, higher expression levels of Lifeact reduced the elongation rate, shrinkage rate and disassembly frequency of actin cables (**Table 1** and **Supplementary Figure S2**), resulting in longer and more stable actin cable structures even during septation (**Figures 2** and **3**). These expression conditions also led to severe abnormalities in hyphal morphology (**Figures 2A,C**). This observation suggests that overexpression of Lifeact and the resulting impairment of the actin dynamics have a high impact on polar growth, such as exocytosis, endocytosis and membrane cytoskeleton interactions for sterol-rich membrane domains at hyphal tips (Takeshita et al., 2012). This would lead to an impaired polar growth and therefore to misshapen hyphal tips. A similar effect of Lifeact stabilizing actin filaments depending on the expression level was shown in plant cells of *Arabidopsis thaliana* (van der Honing et al., 2011; Lemieux et al., 2014). Since tropomyosins are also known to stabilize actin filaments (Bryce et al., 2003), overexpression of TpmA is likely to affect the dynamics of actin cables. Indeed, overexpression of GFP-TpmA also decreased the elongation and shrinkage rates of actin cables (**Supplementary Figure S2**). The different behavior of GFP-TpmA and Lifeact-GFP under the regulation of *alcA* promoter could be explained by the fact that Lifeact and TpmA most likely recognize and bind to different domains of the actin filament. TpmA is an endogenous protein and, therefore, does not interfere with the normal properties of actin filaments at moderate expression levels. In contrast, Lifeact, 17 amino acids of the N-terminus of Abp140 from *S. cerevisiae*, has no homologous sequence in *A. nidulans.* Although there is a homologous gene (AN2190) showing similarity with Abp140 (623 amino acids), the coding protein AN2190 is only 366 amino acids long and lacks the N-terminal Lifeact-like sequence. Lifeact may therefore compete with other actin-binding proteins for its binding domain and alter the binding of other actin-binding proteins, resulting in changed properties of actin filaments under high expression conditions.

Here, we have quantified the elongation rate of actin cables using GFP-TpmA expressed in glycerol medium (elongation rate: 0.58 ± 0.03 μm/s, mean ± SEM) and Lifeact-GFP expressed in threonine + 0.01% glucose medium (elongation rate: 0.43 ± 0.08 μm/s, mean ± SEM, **Table 1**). The elongation rate between GFP-TpmA and Lifeact-GFP did not show statistically significant differences. The elongation rate of 0.58 μm/s by TpmA is comparable with polymerization rates of actin cables in *S. cerevisiae* and *Shizosaccharomyces pombe* (0.3–0.6 μm/s; Yang and Pon, 2002; Huckaba et al., 2004; Johnson et al., 2014). From the comparison of actin cable dynamics among *A. nidulans, S. cerevisiae* and *S. pombe*, the disassembly frequency in *A. nidulans* appears to be higher than that of the yeasts. A typical large-budded yeast cell is ∼10 μm long and has ∼4 μm long actin cables (Moseley and Goode, 2006), whereas in *S. pombe*, the actin cables in interphase often run along the whole long axis of the cell from one pole to the other, resulting in cables longer than 10 μm (Johnson et al., 2014). In contrast, the length of actin cables in *A. nidulans* is usually limited to a range of 2–5 μm, probably due to the higher disassembly frequency.

**FIGURE 8:**
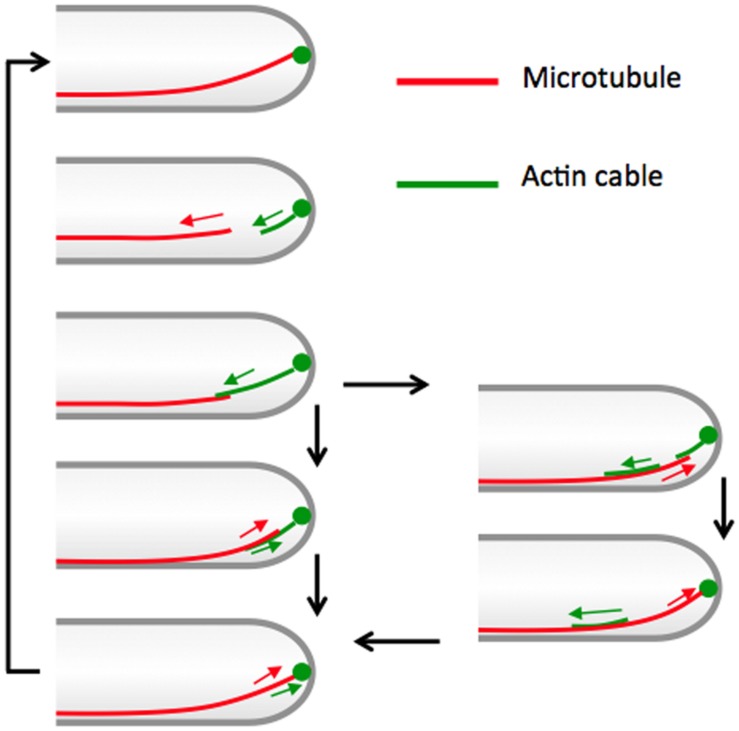
**Working model of coordinated elongation between actin cables and microtubules.** See text for details.

The question arises why the disassembly frequency of actin cables is higher in *A. nidulans* than in the yeasts. Our results from simultaneous visualization of actin cables and microtubules suggest that the assembly and disassembly of actin cables is temporally and spatially associated with microtubule dynamics (**Figures 5** and **6**). Interphase microtubules showed more frequent cycles of polymerization and depolymerization in *A. nidulans* than in the yeasts. In interphase cells of *A. nidulans*, 4–8 microtubules reach the tip cortex per minute (Pöhlmann et al., 2014), whereas microtubules in *S. cerevisiae* play roles in nuclear movement and spindle formation during mitosis (Huisman and Segal, 2005). In interphase cells of *S. pombe*, 3–4 microtubule bundles are arranged along the long axis of the cell. The plus ends of microtubules reach the cell ends and stay there for 1–2 min before exhibiting catastrophe (Tran et al., 2001). The high frequency of microtubules touching the tip cortex in *A. nidulans* is most likely associated with the highly frequent disassembly of actin cables. We propose the following model based on our observations (**Figure 8**). A growing microtubule reaches the hyphal tip cortex where cell-end markers co-localize with the origin of the actin cables (**Figure 4C**; Takeshita et al., 2013). When the microtubule depolymerizes to a subapical region, an actin cable starts to elongate from the tip cortex toward the plus end of the depolymerized microtubule, until they briefly overlap. The shrinkage rate of a microtubule is greater than the elongation rate of actin cables. The microtubule starts to polymerize again along the actin cable to the tip cortex, then the actin cable shrinks. Occasionally, longer actin cables are split into two and the backward actin cable is transported backward along the microtubule (**Figure 8**, right).

According to our model, the question arises why the assembly and disassembly of microtubules and actin cables need to be coordinated in *A. nidulans* but not in the yeasts. One of the reasons may be that both microtubules and actin cables are involved in membrane transport in *A. nidulans*, whereas the actin cables but not microtubules play major roles in vesicle trafficking in yeast (Mishra et al., 2014). In long hyphae, secretory vesicles are delivered to hyphal tips by microtubule-dependent transport and fuse with the tip cortex membrane by actin-dependent myosin V transport (Taheri-Talesh et al., 2008, 2012; Egan et al., 2012; Takeshita et al., 2015). If secretory vesicles need to be transferred from microtubules to actin around the tips, the connection between microtubules and actin cables could guarantee efficient vesicle transfer and secretion. The coordinated polymerization mechanism between microtubules and actin cables possibly contributes to the proper organization of the Spitzenkörper (Harris et al., 2005; Sanchez-Leon et al., 2011; Riquelme and Sanchez-Leon, 2014). The cooperative role of microtubules and actin cables is also suggested in lamellipodia and filopodia formation of mammalian cells (Eltsov et al., 2015).

Moreover, actin cables polymerized from tips also may be involved in microtubule guidance. In *S. cerevisiae*, Kar9 localizing at microtubule plus ends is required for proper alignment of the spindle with the mother-bud axis in an actin cable-myosin dependent manner (Yin et al., 2000). In mammals, tumor suppressor protein adenomatous polyposis coli (APC), which is known as a functional homolog of Kar9, localizes at microtubule plus ends and regulates the microtubule cytoskeleton organization (Gundersen, 2002; McCartney and Nathke, 2008). In addition, APC directly interacts with the formin mDia1 and stimulates actin assembly (Okada et al., 2010). A functional homolog of APC and Kar9 is conserved in *A. nidulans* (Manck et al., 2015), where a similar mechanism might be involved in the regulation of actin cable assembly in a microtubule-dependent manner. The putative mechanism would be a possible explanation for the longer actin cables in the *alpA*-deletion strain (**Figure 7C**).

Although, further analyses are necessary to understand the functional importance and molecular mechanism of the temporal and spatial association between actin cables and microtubules, our results support and supplement our new model of polarized growth (Ishitsuka et al., 2015), where we propose that incessant cycles of assembly and disassembly of the polarity sites are important for maintenance of cell polarity and cell morphology during hyphal growth with highly active exocytosis (Takeshita, 2016). The intracellular Ca^2+^ level is known to regulate actin assembly and vesicle fusion (Janmey, 1994; Schneggenburger and Neher, 2005). Plant root hairs and pollen tubes show an oscillation of the Ca^2+^ concentration at the cell tip, which is correlated with the change in growth rate (Holdaway-Clarke et al., 1997; Monshausen et al., 2008). In mammalian cells, oscillations of cortical actin and Ca^2+^ concentrations correlate with cycles of vesicle secretion (Wollman and Meyer, 2012). Coordinated cycles of cell growth may be a conserved phenomenon shared among various organisms, including filamentous fungi (Lopez-Franco et al., 1994; Ishitsuka et al., 2015), mammalian cells (Wollman and Meyer, 2012) and fission yeast (Das et al., 2012). Our preliminary data show that Ca^2+^ oscillations exist in *A. nidulans* (unpublished data). Therefore, they are likely to play an important role in regulating actin polymerization and triggering the synchronized fusion of accumulated vesicles within a local region for a fine-tuned polarized tip growth of filamentous fungi.

## Author Contributions

NT designed the research project. AB, YI, and NT performed microscopy experiments and analyzed data. NT, ME, and AB prepared sample strains. AB, YI, and GN, and NT wrote the paper with inputs from other coauthors.

## Conflict of Interest Statement

The authors declare that the research was conducted in the absence of any commercial or financial relationships that could be construed as a potential conflict of interest.

## Supplementary Material

FIGURE S1 **Colony, expression, and localization of GFP-TpmA and Lifeact-GFP. (A)** Colonies of wild type (TN02A3), SARB6 (GFP-TpmA), and SNT95 (Lifeact-GFP) grown on minimal medium plus 2% glucose (upper), glycerol (middle), or threonine (bottom) for 3 days at 37°C. **(B)** Quantitative Real-time PCR was made from total RNA of the strains grown in 20 ml minimal medium plus 2% glycerol overnight at 37°C. Error bars represent the standard deviation (*n* = 3). Asterisk represent statistically significant (*p* < 0.01). **(C)** Quantitative Real-time PCR was made from total RNA of SNT95 (Lifeact-GFP) grown in 200 ml minimal medium plus 2% glycerol (dark blue) or 2% threonine plus 0.01% glucose (light blue) liquid culture overnight at 28°C with 180 rpm shaking. Error bars represent the standard deviation (*n* = 3). Asterisk represent statistically significant (*p* < 0.01). **(D)** Relative GFP signal intensity around hyphal tips. SNT95 (Lifeact-GFP) was grown in minimal medium plus 2% glycerol (dark blue) or 2% threonine plus 0.01% glucose (light blue) overnight at 28°C. The GFP signal intensity was measured at the hyphal tips in 3 μm diameter circles by ZEN software (*n* = 20). Asterisks represent statistically significant differences (*p* < 0.01). Hyphal morphologies of the wild type TN02A3 **(E)**, SNT147 (GFP-TpmA; **F**), and SNT149 (GFP-TpmA, Lifeact-mRuby; **G**) strains grown under different conditions (Glc; 2% Glucose, Gly; 2% Glycerol, Thr; 2% Threonine, and Thr plus 0.01% Glc; 2% Threonine plus 0.01% Glucose). Scale bars 1 μm.

FIGURE S2 **Dynamic behavior of actin cables.** Elongation rate **(A)**, shrinkage rate **(B)**, length before disassembly **(C)** of actin cables visualized by GFP-TpmA in minimal medium plus 2% glycerol (red), GFP-TpmA in minimal medium plus 2% threonine (blue), Lifeact-GFP in minimal medium plus 2% glycerol (green), and Lifeact-GFP in minimal medium plus 2% threonine plus 0.01% glucose (purple). **(A)** μm/s (mean ± SEM, *n* = 76, 11, 37, 13), **(B)** μm/s (mean ± SEM, *n* = 100, 17, 43, 13), **(C)** μm (mean ± SD, *n* = 108, 16, 20, 13). Single asterisks represent statistically significant differences (*p* < 0.01). n.s. means no statistically significant differences. **(D)** Catastrophe frequency of actin cable per hyphal tip (green) and frequency of microtubules reaching the hyphal tip (red) per minute. The data are expressed as means ± SD (*n* = 12 and 12, respectively).
