# Supplementary material for: Dynamics of Actin Cables in Polarized Growth of the Filamentous Fungus Aspergillus nidulans
Source: Front Microbiol. 2016 May 9;7:682. doi: 10.3389/fmicb.2016.00682 (PMC4860496; doi:10.3389/fmicb.2016.00682)
Supplement: Supplementary file 1 [file Table_1.DOCX]

Table S1A. *A. nidulans* strains used in this study

| Strain | Genotype | Source |
| --- | --- | --- |
| GR5 | *pyrG89; wA3; pyroA4* | (1) |
| TN02A3 | *pyrG89; argB2;* Δ*nkuA::argB; pyroA4* | (2) |
| RMS011 | *pabaA1; yA; 2*Δ*argB::trpC*Δ*B* | (3) |
| SSH62 | *pyrG89; argB2; ΔnkuA::argB; pyroA4;* [*alcA(p)-mcherry-tubA::pyroA*] | (4) |
| SNT52 | *paba1; pyrG89?; argB2; [teaA(p)*-*mrfp1-teaA::pyr-4]* | (5) |
| SCS13a | *pyrG89; argB2; pyroA4; ΔnkuA::argB; ΔalpA::pyr-4* | (6) |
| SARB6 | *pyrG89; argB2; ΔnkuA::argB; pyroA4; [tpmA(p)-gfp-tpmA::pyr-4]* | This study |
| SNT147 | *pyrG89; argB2; ΔnkuA::argB; pyroA4; [alcA(p)-gfp-tpmA::pyr-4]* | This study |
| SNT95 | *pyrG89; argB2; ΔnkuA::argB; pyroA4; [alcA(p)-lifeact-gfp::pyr-4]* | This study |
| SARB1 | *pyrG89; argB2; ΔnkuA::argB; pyroA4; [alcA(p)-meosthermofp-tpmA::pyr-4]* | This study |
| SARB7 | (SNT147 crossed to RMS011) *pabaA1; pyrG89?;* Δ*argB::trpC*Δ*B?; argB2?; ΔnkuA::argB; [alcA(p)-gfp-tpmA::pyr-4]* | This study |
| SNT148 | *pyrG89; argB2; ΔnkuA::argB; pyroA4; [alcA(p)-lifeact-mruby::pyr-4]* | This study |
| SNT149 | (SARB7 crossed to SNT148) *pyrG89; argB2; ΔnkuA::argB; [alcA(p)-gfp-tpmA::pyr-4][alcA(p)-lifeact-mRuby::pyr-4]* | This study |
| SNT150 | (SNT52 crossed to SNT95) *pyrG89?; argB2; ΔnkuA::argB;*  *[alcA(p)-lifeact-gfp::pyr-4] [teaA(p)*-*mrfp1-teaA::pyr-4]* | This study |
| SARB44 | (SSH62 crossed to SARB6) *pyrG89; argB2; ΔnkuA::argB; pyroA4; [alcA(p)-gfp-tpmA::pyr-4][alcA(p)-mcherry-tubA::pyroA]* | This study |
| SNT99 | (SSH62 crossed to SNT95) *pyrG89; argB2; ΔnkuA::argB; pyroA4; [alcA(p)-lifeact-gfp::pyr-4][alcA(p)-mcherry-tubA::pyroA]* | This study |
| SNT152 | (SCS13a crossed to SARB7) *pyrG89; pyrG89?;* Δ*argB::trpC*Δ*B?; argB2?; ΔnkuA::argB; [alcA(p)-gfp-tpmA::pyr-4] ; ΔalpA::pyr-4* | This study |
| SNT152 | *pyrG89; argB2; ΔnkuA::argB; pyroA4; [alcA(p)-cameleon::pyr-4]* | This study |

All strains carry *veA1* mutation.

Table S1B. Plasmids used in this study.

| Plasmid | Description | Reference or source |
| --- | --- | --- |
| pEGFP-N1-Lifeact | lifeact sequence cloned in pEGFP-N1 (Clonetech) | (7) |
| pmRFPRuby-N1-Lifeact | lifeact sequence cloned in pmRFPRuby-N1 (Clonetech) | Rusty Lansford lab |
| AAV-6P-SEW-YC3.6 | Cameleon sequence cloned in AAV-6P-SEWB | (8) |
| pYH27 | *alcA(p)-gfp-tpmA::pyr-4* | this study |
| pARB7 | *tpmA(p) -gfp-tpmA::pyr-4* | this study |
| pARB1 | *alcA(p)-meosfpthermo-tpmA::pyr-4* | this study |
| pNT52 | *alcA(p)-lifeact-egfp::pyr-4* | this study |
| pNT51 | *alcA(p)-lifeact-mruby::pyr-4* | this study |
| pARB10 | *alcA(p)-cameleon::pyr-4* | this study |

Table S1C. Primers used in this study.

| Name | Sequence (5‘-3‘) |
| --- | --- |
| TpmA_fw | GGCGCGCCCATGGACAGAATCAAGGAG |
| TpmA_rev | TTAATTAAACACTGTTCAAGGAG |
| (p)tpmA_for | CGAGAATTCACCGGCCTGGTCGAAAT |
| (p)tpmA_rev | CATGGTACCGGTGGAGGGCTAGCAGC |
| Eos_KpnI_fwd | TGGTACCATGAGTGCGATTAAGCCAGAC |
| mEos_AscI_rev | TGGCGCGCCCCGTCTGGCATTGTCAGGC |
| lifeact-f-kpn | GGTACCATGGGAGTAGCAGATCTAAT |
| GFP_sto_PacI_rev | TTAATTAAattTTTGTATAGTTCATCCATGCC |
| lifeact_mRuby_f2 | CATGGTACCATGGGAGTAGCAGATCTAAT |
| lifeact_mRuby_rev_PacI | TCATTAATTAATCACCCTCCGCcCAGG |
| Cameleon-f-kpn | GGTACCGTCGAATTCAAGCTGCTAGC |
| Cameleon-r-pac | TTAATTAATGATTATCGATAAGCTTGCG |
| H2B FW-qRT | CTGCCGAGAAGAAGCCTAGCAC |
| H2B Rev-qRT | GAAGAGTAGGTCTCCTTCCTGGTC |
| qRT_GFP_fw | GGCCAACACTTGTCACTACT |
| qRT_GFP_rev | CACGTGTCTTGTAGTTCCC |

References in Table S1A-C.

1. **Waring RB, May GS, Morris NR.** 1989. Characterization of an inducible expression system in *Aspergillus nidulans* using alcA and tubulin-coding genes. Gene **79:**119-130.

2. **Nayak T, Szewczyk E, Oakley CE, Osmani A, Ukil L, Murray SL, Hynes MJ, Osmani SA, Oakley BR.** 2006. A versatile and efficient gene-targeting system for *Aspergillus nidulans*. Genetics **172:**1557-1566.

3. **Stringer MA, Dean RA, Sewall TC, Timberlake WE.** 1991. Rodletless, a new *Aspergillus* developmental mutant induced by directed gene inactivation. Genes Dev **5:**1161-1171.

4. **Takeshita N, Mania D, Herrero de Vega S, Ishitsuka Y, Nienhaus GU, Podolski M, Howard J, Fischer R.** 2013. The cell end marker TeaA and the microtubule polymerase AlpA contribute to microtubule guidance at the hyphal tip cortex of *Aspergillus nidulans* for polarity maintenance. J Cell Sci **126:** 5400-5411.

5. **Takeshita N, Higashitsuji Y, Konzack S, Fischer R.** 2008. Apical sterol-rich membranes are essential for localizing cell end markers that determine growth directionality in the filamentous fungus *Aspergillus nidulans*. Mol Biol Cell **19:**339-351.

6. **Enke C, Zekert N, Veith D, Schaaf C, Konzack S, Fischer R.** 2007. *Aspergillus nidulans* Dis1/XMAP215 protein AlpA localizes to spindle pole bodies and microtubule plus ends and contributes to growth directionality. Eukaryot Cell **6:**555-562.

7. **Riedl J, Crevenna AH, Kessenbrock K, Yu JH, Neukirchen D, Bista M, Bradke F, Jenne D, Holak TA, Werb Z, Sixt M, Wedlich-Soldner R.** 2008. Lifeact: a versatile marker to visualize F-actin. Nat Methods **5:**605-607.

8. **Minderer M, Liu W, Sumanovski LT, Kugler S, Helmchen F, Margolis DJ.** 2012. Chronic imaging of cortical sensory map dynamics using a genetically encoded calcium indicator. J Physiol **590:**99-107.
