# Supplementary figures and images for: Dynamics of Actin Cables in Polarized Growth of the Filamentous Fungus Aspergillus nidulans
Source: Front Microbiol. 2016 May 9;7:682. doi: 10.3389/fmicb.2016.00682 (PMC4860496; doi:10.3389/fmicb.2016.00682)

Fig. S1

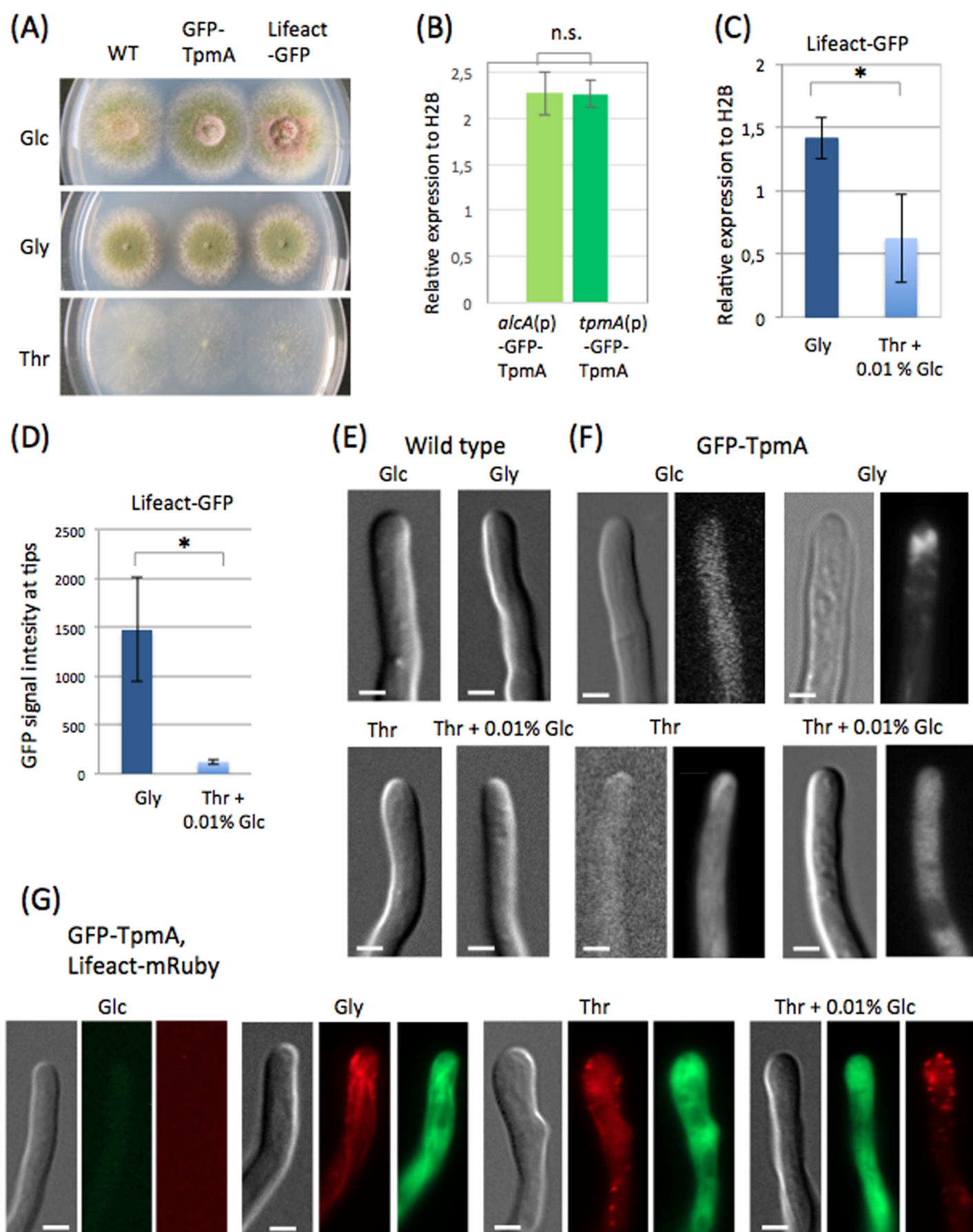

Fig. S2

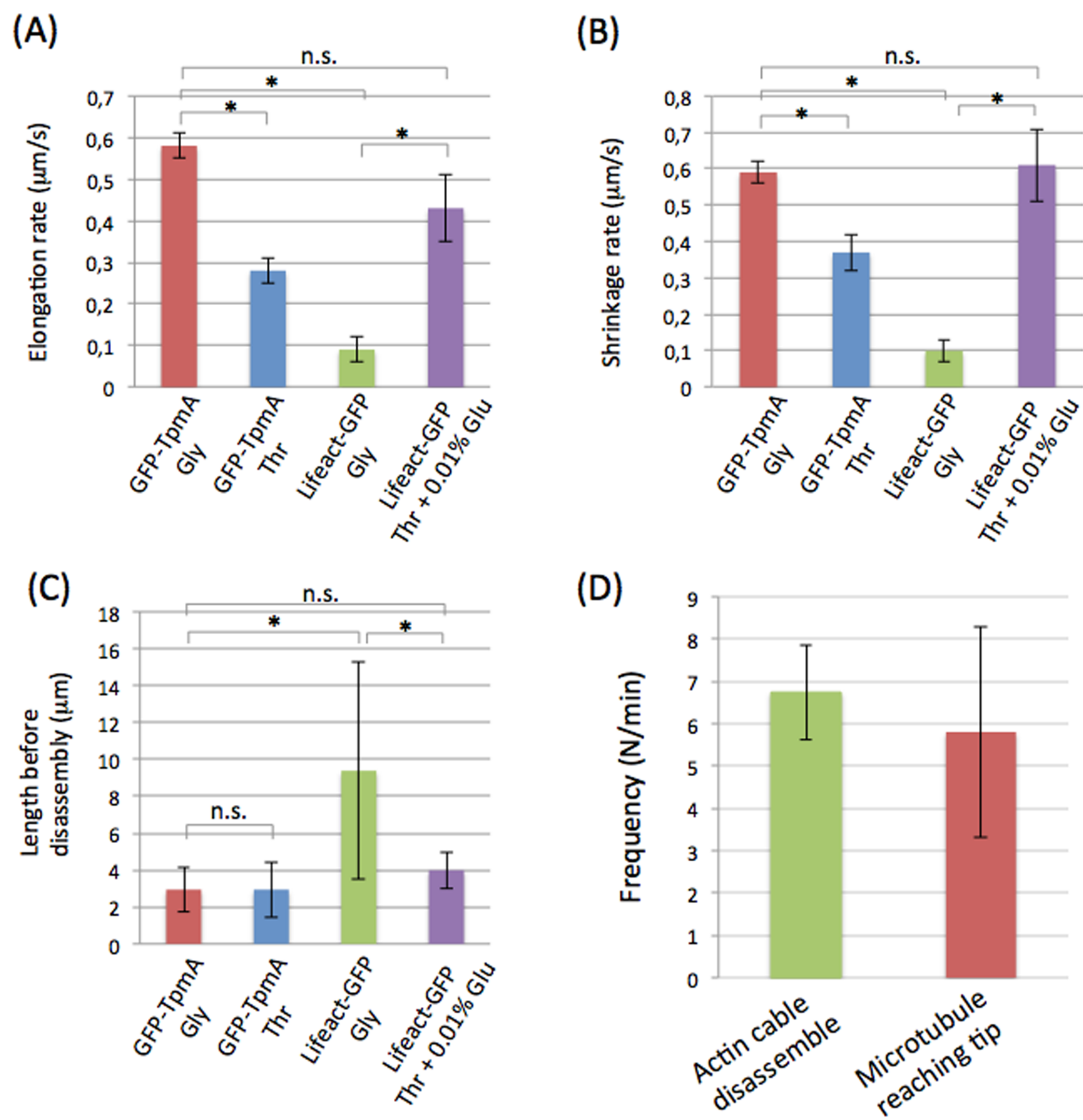

Supplement: FIGURE S1 — Colony, expression, and localization of GFP-TpmA and Lifeact-GFP. (A) Colonies of wild type (TN02A3), SARB6 (GFP-TpmA), and SNT95 (Lifeact-GFP) grown on minimal medium plus 2% glucose (upper), glycerol (middle), or threonine (bottom) for 3 days at 37°C. (B) Quantitative Real-time PCR was made from total RNA of the strains grown in 20 ml minimal medium plus 2% glycerol overnight at 37°C. Error bars represent the standard deviation (n = 3). Asterisk represent statistically significant (p < 0.01). (C) Quantitative Real-time PCR was made from total RNA of SNT95 (Lifeact-GFP) grown in 200 ml minimal medium plus 2% glycerol (dark blue) or 2% threonine plus 0.01% glucose (light blue) liquid culture overnight at 28°C with 180 rpm shaking. Error bars represent the standard deviation (n = 3). Asterisk represent statistically significant (p < 0.01). (D) Relative GFP signal intensity around hyphal tips. SNT95 (Lifeact-GFP) was grown in minimal medium plus 2% glycerol (dark blue) or 2% threonine plus 0.01% glucose (light blue) overnight at 28°C. The GFP signal intensity was measured at the hyphal tips in 3 μm diameter circles by ZEN software (n = 20). Asterisks represent statistically significant differences (p < 0.01). Hyphal morphologies of the wild type TN02A3 (E), SNT147 (GFP-TpmA; F), and SNT149 (GFP-TpmA, Lifeact-mRuby; G) strains grown under different conditions (Glc; 2% Glucose, Gly; 2% Glycerol, Thr; 2% Threonine, and Thr plus 0.01% Glc; 2% Threonine plus 0.01% Glucose). Scale bars 1 μm. [file Presentation_1.PDF]
